# Quantification of mutant SPOP proteins in prostate cancer using mass spectrometry-based targeted proteomics

**DOI:** 10.1186/s12967-017-1276-7

**Published:** 2017-08-15

**Authors:** Hui Wang, Christopher E. Barbieri, Jintang He, Yuqian Gao, Tujin Shi, Chaochao Wu, Athena A. Schepmoes, Thomas L. Fillmore, Sung-Suk Chae, Dennis Huang, Juan Miguel Mosquera, Wei-Jun Qian, Richard D. Smith, Sudhir Srivastava, Jacob Kagan, David G. Camp, Karin D. Rodland, Mark A. Rubin, Tao Liu

**Affiliations:** 1 Biological Sciences Division, Pacific Northwest National Laboratory, P.O. Box 999, MSIN: K8-98, Richland, WA 99354 USA; 2 Institute of Precision Medicine of Weill Cornell Medical College and New York Presbyterian Hospital, New York, NY USA; 3 Division of Cancer Prevention, Cancer Biomarkers Research Group, National Cancer Institute, Bethesda, MD USA

**Keywords:** Mass spectrometry, Targeted proteomics, SPOP mutation, Prostate cancer, Biomarker, PRISM-SRM

## Abstract

**Background:**

Speckle-type POZ protein (SPOP) is an E3 ubiquitin ligase adaptor protein that functions as a potential tumor suppressor, and SPOP mutations have been identified in ~10% of human prostate cancers. However, it remains unclear if mutant SPOP proteins can be utilized as biomarkers for early detection, diagnosis, prognosis or targeted therapy of prostate cancer. Moreover, the SPOP mutation sites are distributed in a relatively short region with multiple lysine residues, posing significant challenges for bottom-up proteomics analysis of the SPOP mutations.

**Methods:**

To address this issue, PRISM (high-pressure, high-resolution separations coupled with intelligent selection and multiplexing)-SRM (selected reaction monitoring) mass spectrometry assays have been developed for quantifying wild-type SPOP protein and 11 prostate cancer-derived SPOP mutations.

**Results:**

Despite inherent limitations due to amino acid sequence constraints, all the PRISM-SRM assays developed using Arg-C digestion showed a linear dynamic range of at least two orders of magnitude, with limits of quantification ranged from 0.1 to 1 fmol/μg of total protein in the cell lysate. Applying these SRM assays to analyze HEK293T cells with and without expression of the three most frequent SPOP mutations in prostate cancer (Y87N, F102C or F133V) led to confident detection of all three SPOP mutations in corresponding positive cell lines but not in the negative cell lines. Expression of the F133V mutation and wild-type SPOP was at much lower levels compared to that of F102C and Y87N mutations; however, at present, it is unknown if this also affects the biological activity of the SPOP protein.

**Conclusions:**

In summary, PRISM-SRM enables multiplexed, isoform-specific detection of mutant SPOP proteins in cell lysates, providing significant potential in biomarker development for prostate cancer.

**Electronic supplementary material:**

The online version of this article (doi:10.1186/s12967-017-1276-7) contains supplementary material, which is available to authorized users.

## Background

Cancer is considered to be a genomic disease [1], and recent studies have identified mutations in speckle-type POZ protein (SPOP) in up to 15% of human primary prostate cancer (PCa) patients [2, 3]. SPOP mutations represent a distinct subclass of PCa, in that tumors with SPOP mutations generally lack other common features of PCa, including the PCa-specific TMPRSS2 and ETS family gene rearrangements (present in ~50% of PCa), abnormalities in the phosphatidylinositide 3-kinase pathway, or deletion and mutations of the tumor suppressor gene TP53 [2, 4–6]. Therefore, the presence of mutant SPOP may serve as a specific protein biomarker for early detection of prostate carcinogenesis, particularly in patients lacking the TMPRSS2-ETS gene fusions. The qualitative and quantitative study of mutant SPOP proteins can contribute to improved understanding of the functional significance and molecular mechanism of SPOP mutations in prostate cancer, and their relevance for targeted cancer therapies in clinical applications [7–11].

Currently available techniques for identification of SPOP mutation are mainly genomic methods, including whole-exome sequencing, Sanger sequencing, and quantitative RT-PCR [2, 12]. Although there have been several studies of the effects of forced over-expression of mutant SPOP protein in immortal cell lines [9–11], there appears to be no studies of endogenous SPOP mutations at the protein level in either primary prostate cancer cell lines or prostate cancer tissues, primarily due to the lack of antibodies capable of distinguishing between mutant and wild type SPOP protein. Traditional methods for protein measurement, which rely heavily on antibodies or other affinity reagents, are problematic for detection of SPOP mutations, for multiple reasons. First, most SPOP mutations in PCa are missense mutations, in which only one amino acid is altered, so specific antibodies against the mutant epitopes are required to distinguish the mutant isoforms from the wild type SPOP protein. This is a difficult task, even in such well-studied missense mutant proteins as TP53 and KRAS [13]. Also, various mutations can occur in the same amino acid position; for example, the most frequently mutated residue of SPOP, F133, displays four types of variants (F→V/L/C/S), making the antibody development much more difficult [2]. Moreover, seven amino acid (AA) residues covering the 11 most frequent mutations are clustered in a 49-AA region in SPOP, making it extremely difficult to develop mutation-specific antibodies in such an area. In this report we demonstrate that a highly sensitive targeted proteomic method, PRISM-SRM (high-pressure, high-resolution separations coupled with intelligent selection and multiplexing—selected reaction monitoring), is capable of providing quantitative information on both wild-type (WT) SPOP and four distinct single amino acid variants of SPOP that have been implicated in prostate cancer.

Mass spectrometry (MS)-based targeted proteomics methods such as SRM have become an increasingly important strategy for detection and quantitation of proteins in complex biological samples [14–18]. Combined with heavy isotope-labeled internal standards, targeted MS can provide highly sensitive and precise quantification of protein isoforms with a difference as small as a single AA residue. An example of this approach is the use of SRM-MS to identify and quantify multiple mutations of the Ras protein, either with enrichment by immunoprecipitation [13, 19] or SDS-PAGE [20]. Compared to Ras mutations where only two adjacent AA residues are involved, the most frequent SPOP mutations are distributed at seven distinct positions within a 49-AA region (AA 87–135) [2], requiring multiple distinct probes to fully characterize mutant SPOP proteins. Moreover, there are multiple lysine residues in this region, producing very small tryptic peptides including the mutated region that are unsuitable for bottom-up proteomics analysis. Herein, we describe the development of new SPOP mutation-specific SRM assays for highly sensitive and multiplexed quantification of multiple SPOP protein isoforms using combined Arg-C digestion and PRISM-SRM. The PRISM-SRM method developed at our laboratory provides both increased loading and reduced complexity, leading to higher sensitivity and specificity for low abundance protein quantification. It has been successfully applied to the quantitative analysis of several PCa-associated biomarkers, such as prostate-specific antigen (PSA) [21, 22], AGR2 [23] and TMPRSS2-ERG fusion proteins [5, 24]. The validity of these PRISM-SRM assays for quantifying low abundance SPOP proteins was demonstrated using several SPOP mutant cell lines, providing an effective method for verifying the mutant SPOP proteins as biomarkers in PCa.

## Methods

### Materials

Purified peptides (>97% purity) isotopically labeled with C-terminal [^13^C_6_^15^N_2_] lysine or [^13^C_6_^15^N_4_] arginine and their unlabeled counterparts were synthesized by Thermo Fisher Scientific (San Jose, CA). Urea, dithiothreitol (DTT), iodoacetamide (IAA) and formic acid (FA) were purchased from Sigma-Aldrich (St. Louis, MO). Sequencing grade endoproteinases Arg-C and Asp-N were purchased from Roche (Indianapolis, IN). Recombinant SPOP protein was purchased from OriGene (Rockville, MD). The VCaP prostate cancer cells were obtained from the American Type Culture Collection (Manassas, VA).

### SPOP cell lines

Wild-type (WT) and three SPOP mutants (Y87N, F102C and F133V) cell lines were established by transfecting human embryonic kidney (HEK) 293T cells with expression constructs encoding either WT SPOP or specific mutant SPOP sequences [2, 3].

### Protein extraction and digestion

The cell line samples were lysed in 8 M urea and 50 mM NH_4_HCO_3_ with vortexing and sonication (3 × 20 s), and the protein concentration was estimated using BCA assay (Pierce, Rockford, IL). Proteins were reduced with 10 mM DTT at 37 °C for 1 h, and subsequently alkylated with 40 mM IAA at room temperature for 1 h in the dark. After tenfold dilution with 100 mM Tris–HCl buffer (pH 7.6), 500 mM CaCl_2_ was added to the sample to reach a final concentration of 1 mM. Protein digestion was performed at an enzyme-to-protein ratio of 1:50, at 37 °C for 3 h. The enzymatic reaction was stopped by adding trifluoroacetic acid (TFA) solution to a final concentration of 0.1%. The resulting peptide digests were then cleaned up by C18 SPE cartridge (Supelco, Bellefonte, PA). The recombinant SPOP protein sample was digested in the same way without the lysis step.

### LC–MS/MS analysis

LC–MS/MS analyses of the Arg-C and Asp-N digests of recombinant SPOP protein were carried out on a nanoACQUITY UPLC (Waters, Milford, MA) coupled to an Orbitrap Velos mass spectrometer (Thermo Scientific). The LC separation was performed on a C18 column with 0.1% FA in H_2_O/ACN as buffer A/B at a gradient of 1–8% B in 0–2 min, 8–12% B in 2–20 min, 12–30% B in 20–75 min, 30–45% in 75–97 min, and 45–95% B in 97–100 min. The heated capillary temperature and spray voltage was 350 °C and 2.2 kV, respectively. Full MS spectra were recorded at a resolution of 60,000 over the range of *m/z* 300–2000. Ten parent ions with most abundant intensity were selected for MS/MS using CID with a collision energy of 35V. The resulting raw data were searched against the UniProt human protein database [25] using the MSGF+ algorithm [26, 27]. The parameter was 20 ppm tolerance for precursor ion masses and 0.5 Da tolerance for fragment ions, with static carbamidomethylation on cysteine residues (+57.0215 Da) and dynamic oxidation of methionine residues (+15.9449 Da). The data filtering criteria is MSGF E < 10^−8^, Q < 0.01 and mass measurement error <20 ppm (±10 ppm).

### PRISM fractionation

The PRISM pre-fractionation method has been described previously [21]. Briefly, peptide fractionation was performed on an in-house C18 (3 µm, 300 Å, Jupiter, Phenomenex, Torrance, CA) packed capillary column (200 µm i.d. ×40 cm) at a flow rate 2.2 µL/min on a Waters nanoACQUITY UPLC system. Buffers for high and low pH reversed-phase (RP) fractionation were 10 mM of ammonium formate (pH 9) in H_2_O/90% ACN (A/B), 0.1% FA in H_2_O/ACN (A/B) and 0.1% TFA in H_2_O/ACN (A/B), respectively. The LC gradient was 0.5% B in 0–35 min, 0.5–20% B in 35–37 min, 20–55% B in 37–107 min and 55–90% B in 132–135 min. Forty-five micrograms of protein digests were injected for each PRISM run. The 1-min fractions were collected in vials of a 96-well plate and diluted to 20 µL with water for the next dimensional LC-SRM analysis.

### SRM assay development

The SRM transitions of each target peptide were optimized separately in direct infusion mode on a TSQ Vantage triple quadrupole mass spectrometer (Thermo Fisher Scientific). The predominant charge states of a peptide were selected as parent ions. Then, collision energy (CE) was ramped for each charged precursor to get the most intense fragments. Optimal CE value was determined by calculating the average value of three CE measurements. The transitions and optimal CE values were further validated by LC-SRM analysis of the heavy peptide standards spiked in a VCaP digest. Transition with low MS signal and high interference level were removed.

The method for determination of the calibration curve and the definition of the limit of detection (LOD) and limit of quantification (LOQ) has been described previously [5], with the addition of Arg-C digests from VCaP cell line at a final concentration of 1 µg/µL as a matrix, as described below. The reverse response curve for each SPOP peptide was generated by varying the amount of heavy peptide with a constant amount of light peptide (5 fmol/µL for the peptide in the non-mutant region, 10 fmol/µL for peptides covering potential mutant site F102 or F133 and 20 fmol/µL for a peptide including possible mutant site Y87). The twelve-point response curves (0.05, 0.1, 0.25, 0.5, 1, 2.5, 5, 10, 25, 50, 100 and 250 fmol/µL for heavy peptides in non-mutant region, and twofold and fourfold of each concentration data point for heavy peptides containing mutation site F102/F133 and Y87, respectively) covered more than 2 orders of magnitude. Three replicates of each data point were analyzed.

### LC-SRM analysis

All LC-SRM experiments were performed on a nanoACQUITY UPLC coupled to TSQ Vantage MS instrument, with 4.5 µL of sample injection for each measurement. 0.1% FA in water and 0.1% in 90% ACN were used as buffer A and B, respectively. Peptide separations were performed by an in-house C18 (3 µm, 300 Å, Jupiter) packed capillary column (75 µm i.d. ×40 cm) at a flow rate of 350 nL/min using gradient of 0.5% B in 0–14.5 min, 0.5–15% B in 14.5–15.0 min, 15–40% B in 15–30 min and 45–90% B in 30–32 min. The heated capillary temperature and spray voltage was set at 350 °C and 2.4 kV, respectively. Both the Q1 and Q3 were set as 0.7 FWHM. A scan width of 0.002 *m/z* and a dwell time of 10 ms were used. All the SRM data were analyzed by Skyline Software [28].

## Results

### Peptide selection

**Fig. 1:**
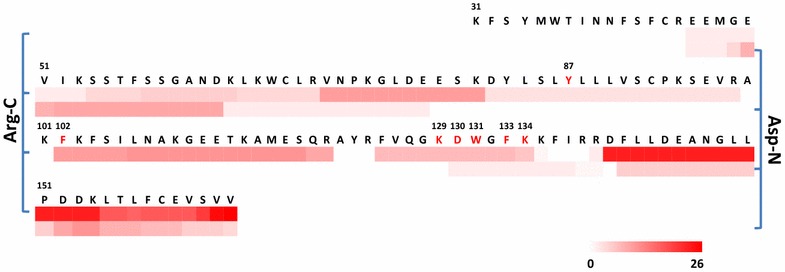
Heat map of spectral counts of the peptides generated from recombinant SPOP protein MATH domain by Arg-C and Asp-N digestion. The AAs shown in *red* and *underlined fonts* represent the reported potential mutant sites. The sequence of SPOP is obtained from UniProt [25]. The heat map for spectral counts of peptides generated from full-length SPOP protein shown in Additional file 2: Figure S1

In contrast to wild-type proteins, peptide selection for mutant proteins is constrained to those peptides that cover the mutation sites. Consequently, some candidate peptides may not meet the “gold standard”, e.g., 6–20 AA in length, good LC elution, and strong MS signal, which is typically considered for selection of target peptides. The most frequent SPOP mutations in prostate cancer are located at seven distinct AA residues (e.g., Y87, F102, W131 and F133) clustered in the MATH domain (AA 31–166) [2, 29]. The AA sequence of SPOP has eight lysine (K) and three arginine (R) residues between the first (Y87) and last mutation site (K134) in the MATH domain (shown in Fig. 1). Use of trypsin for digestion of SPOP is therefore impractical for LC-SRM analysis as most of the resulting tryptic peptides that include the mutation sites are too short. Short peptides often have weak retention on commonly used C18 RPLC column, and the choice of transitions for short peptides is much more limited (i.e., lower specificity). Additional file 1: Table S1 shows the peptide candidates covering the seven potential SPOP mutation sites generated by in silico digestion. Six of seven tryptic peptides have peptide length of less than 6 AA (Additional file 1: Table S1). Lys-C digestion has similar limitations due to a large number of lysine residues in this region.

Arg-C, on the other hand, specifically hydrolyzes proteins and peptide bonds at the C-terminal side of arginine residues; unlike trypsin, Arg-C does not cleave at the C-terminal of lysine residues. The result of in silico digestion indicates that the Arg-C peptides can cover the majority of the SPOP mutation sites (Additional file 1: Table S1). Besides Arg-C, Asp-N which cleaves peptide bonds at the N-terminal of aspartic acid (D) or glutamic acid (E) residues was also considered. All the Asp-N peptides covering the mutation sites are appropriate in length for SRM measurement (Additional file 1: Table S1). To determine which enzymatic digestion generates more MS-responsive peptides that cover the mutation sites, these two proteases were used to digest recombinant SPOP protein, followed by LC–MS/MS analysis of the digests. Additional file 2: Figure S1 shows a heat map of the spectral counts of SPOP peptides generated by the two enzymatic digestions. For both conditions, the identification of peptides in the mutation region was more difficult compared to the non-mutation areas. The sequence coverage and relative peptide abundance by Arg-C in the entire protein, especially in the mutation region (Fig. 1), is much higher than that of Asp-N digestion; Arg-C also produced a total of 162 unique peptides, compared to 86 by Asp-N, demonstrating the superior performance of Arg-C digestion for producing surrogate peptides for the SPOP protein isoforms. Therefore, Arg-C was selected for SPOP proteolysis, and PRISM-SRM assays for a total of 16 SPOP peptide candidates were developed, including 11 mutation sites (Y87C/N, F102C, K129C, D130H, W131C/G, F133L/V/S and K134N) [2] and their related WT counterparts, as well as two “normal” peptides shared by all protein isoforms in the non-mutation region.

### Transition selection and validation

Typically, the most intensive and interference-free precursor and fragment ions are selected as the transitions for the target peptides. However, transition selection for mutant proteins can be challenging, due to the high similarity in AA sequence between the point mutation peptides and its normal form, such that they generate similar series of fragment ions, especially if the mutation site is near the C- or N-terminus. In this situation, distinguishing between mutant and normal peptides relies on the precursor *m/z*; if their precursor *m/z* values are also close, then additional information, such as the difference in LC elution time, is also needed.

Most of the Arg-C peptides covering the SPOP mutation site(s) have multiple internal lysine residues, and hence carry multiple charges with the 4+ precursor ions being the most intense (Table 1). Compared to the common doubly charged precursors, the m/z differences between mutant and normal precursors with 4+ charges are much smaller. For instance, the mutant peptide VNPKGLDEESKDYLSLCLLLVSCPKSEVR and its WT peptide VNPKGLDEESKDYLSLYLLLVSCPKSEVR only have a small *m/z* difference of 0.7581, and share many intensive fragment ions, which is insufficient to distinguish them solely by the SRM transitions. In our LC-SRM analysis of the peptide mixture, we found two peaks appeared in the extracted ion chromatogram (XIC) of peptide VNPKGLDEESKDYLSLYLLLVSCPKSEVR (Peaks 1 and 2 in Additional file 3: Figure S2A), which was caused by co-scanning of the WT peptide and its mutant counterpart VNPKGLDEESKDYLSLCLLLVSCPKSEVR. On the other hand, only Peak 1 was detected in the XIC of VNPKGLDEESKDYLSLCLLLVSCPKSEVR (Additional file 3: Figure S2B), suggesting that Peak 1 belongs to this mutant peptide while Peak 2 belongs to the WT peptide VNPKGLDEESKDYLSLYLLLVSCPKSEVR. The final validated, optimal transitions for the 16 target peptides are listed in Additional file 4: Table S2.

**Table 1:** LOD and LOQ (fmol/µg of total protein) of the PRISM-SRM assays for WT and mutant SPOP proteins

Peptide	Charge	Transition	Production type	LOD fmol/µg	LOQ fmol/µg	t_R_ min
VNPKGLDEESKDYLSLYLLLVSCPKSEVR	4+	838.7−962.2	y8	0.4	1	30.0
VNPKGLDEESKDYLSLNLLLVSCPKSEVR	4+	826.4−962.5	y8	0.2	0.4	28.4
AKFKFSILNAKGEETKAMESQR	4+	629.1−721.3	y6	0.5	1	24.5
AKCKFSILNAKGEETKAMESQR	4+	632.3−721.3	y6	0.5	1	23.8
FVQGKDWGFKKFIR	4+	439.8−895.6	y7	0.2	0.5	25.6
FVQGKDWGVKKFIR	4+	427.8−847.6	y7	0.1	0.2	24.5
LADELGGLWENSR	2+	730.4−918.4	y8	0.05	0.1	27.9
SLASAQCPFLGPPR	2+	750.9−943.5	y8	0.05	0.1	26.1

*t*_*R*_ retention time

### Matrix effects on peptide loss

During the transition validation, we also noted that some hydrophobic SPOP peptides showed a poor response in the LC-SRM analysis of the neat peptide mixture. One of the examples is that peptide VNPKGLDEESKDYLSLYLLLVSCPKSEVR and its two mutant forms VNPKGLDEESKDYLSLCLLLVSCPKSEVR and VNPKGLDEESKDYLSLNLLLVSCPKSEVR were barely detected in the neat peptide mixture (Additional file 5: Figure S3A). We postulated that the lack of a protective matrix in the pure peptide stocks might contribute to substantial loss of the highly hydrophobic peptides. To test this hypothesis, we prepared the heavy peptide stock solutions in a VCaP cell digest with a final concentration of 0.2 µg/µL and repeated the analysis. Indeed, all three heavy peptides from the stock solution with matrix were detected with much better response (Additional file 5: Figure S3B).

The matrix effect on all the 16 target peptides was further investigated by plotting the peptide recovery (peak area ratio of no-matrix versus with-matrix) to their theoretical hydrophobicity calculated based on AA sequence using the Thermo Fisher on-line peptide analyzing tool (http://www.thermofisher.com; Additional file 6: Figure S4). Overall, three of the peptides with hydrophobicity <30 showed high recovery (>70%); six peptides with hydrophobicity >32.5 (color zone) have ~10% recovery; three peptides with hydrophobicity >40 (the examples in Additional file 3: Figure S2) have only 2% recovery. This result demonstrated that adding a matrix with a similar peptide concentration level for LC-SRM analysis (0.2 µg/µL) to the pure peptide stocks is an effective way to reduce peptide loss, especially for the hydrophobic peptides. Moreover, it also helped to reduce run-to-run variation, e.g., the CV of H/L ratio of peptides reduced from 4 to 34% using no-matrix stocks (mean CV ~14%) to <10% using the stocks with matrix (mean CV ~4%), demonstrating the importance of matrix on the stock stability of these Arg-C SPOP peptides. Two other matrices, tryptic digests of LNCaP prostate cancer cells and *Shewanella oneidensis*, were also tested and they were equally effective in reducing the loss of hydrophobic peptides and improving the reproducibility of LC-SRM analysis (data not shown).

### Peptide loss in PRISM fractionation

Regular LC-SRM could not detect many of the low abundance SPOP proteins in the cells (see Additional file 7: Figure S5). Therefore, PRISM-SRM, a highly sensitive targeted quantification technique which has enabled quantification of low-abundance proteins at pg/mL level in depleted plasma/serum or low 100 s copies per cell levels [30], was used for developing the SPOP mutation assays. Compared to regular LC-SRM, PRISM-SRM allows for up to 50 times more loading (maximum 50 µg) of samples in the first dimension high pH (pH 9) reversed-phase LC separation (with optional concatenation for multiplexing), specific target peptide-containing fractions with much-reduced sample complexity are then analyzed by LC-SRM, leading to as high as 100 times improvement in sensitivity [21].

However, not all peptides can take full benefit of the PRISM separation (e.g., due to potential peptide loss in the additional dimension of separation). We evaluated the recovery of the peptides after fractionation by comparing the heavy peptide intensity of PRISM-SRM to that of straight LC-SRM analysis. It was noted that all the peptides in the mutant region had a PRISM-SRM/LC-SRM ratio of ≤1. Three peptides with significant sample loss have p*I* of ~10 or higher (Additional file 8: Figure S6). We reason that the sample loss may be caused by the strong interaction between the positively charged basic peptides and the negatively charged silanol groups on the separation media in the high pH separation condition (pH 9). To determine if acidic RP conditions can reduce the PRISM peptide loss, we tested 0.1% FA and 0.1% TFA in H_2_O/ACN as the mobile phases A/B for the first-dimension PRISM fractionation. Indeed, the results showed that FA mobile phases slightly improved the overall peptide detection, while TFA mobile phases provided a significant boost of peptide signals, in particular for the peptides having high p*I* values (Additional file 8: Figure S6). Thus, 0.1% TFA mobile phases were selected for the optimal condition of SPOP PRISM fractionation. Overall, PRISM-SRM of these long, multiple-internal-lysine-containing Arg-C peptides did not provide even higher (e.g., >100×) improvement in sensitivity like the case of other “regular” peptides, presumably due to the reduced matrix protection in the PRISM fractions (i.e., the majority of the non-target peptides are removed) and the reduced orthogonality with the second-dimension acid RPLC separation. However, it still enabled much needed more sensitive measurements of the SPOP mutation peptides comparing to regular straight LC-SRM analysis.

### PRISM-SRM quantification

**Fig. 2:**
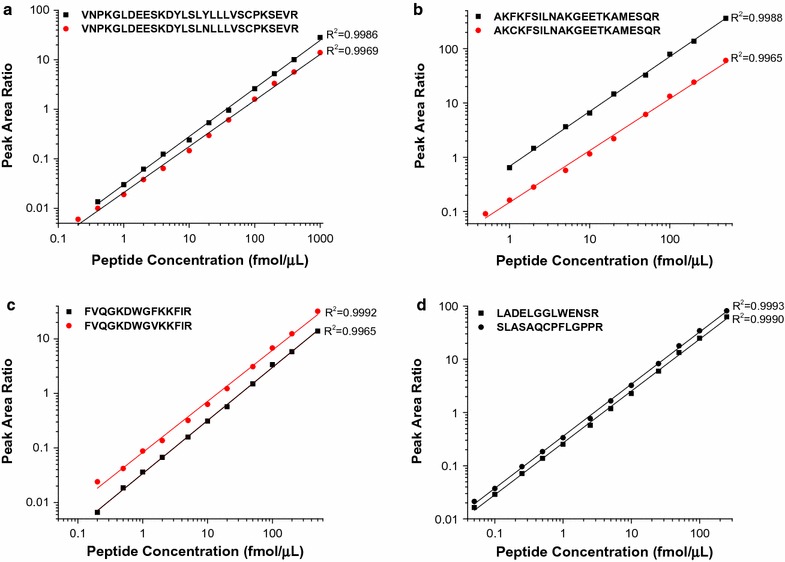
The response curve of the PRISM-SRM assays (in log–log scale) for eight SPOP peptides (*black* WT; *red* mutant): VNPKGLDEESKDYLSLYLLLVSCPKSEVR/VNPKGLDEESKDYLSLNLLLVSCPKSEVR (**a**), AKFKFSILNAKGEETKAMESQR/AKCKFSILNAKGEETKAMESQR (**b**), FVQGKDWGFKKFIR/FVQGKDWGVKKFIR (**c**) and LADELGGLWENSR/SLASAQCPFLGPPR (**d**)

To quantify the mutant SPOP peptides, the response curve, LOD and LOQ for eight SPOP peptides (three peptides represented as both WT and mutant versions, plus the two “common” peptides in the non-mutated region) in PRISM-SRM assay was determined. The result was shown in Fig. 2 and Table 1. Although the Arg-C digested SPOP peptides in the mutation region are longer and more hydrophobic, the mutation PRISM-SRM assays still showed an excellent linear dynamic range of more than 2 orders of magnitude (Fig. 2). The LOD and LOQ for SPOP peptides range from 0.05 to 0.5 and 0.1 to 1.0 fmol/µg, respectively. The median CV for each peptide in triplicate PRISM-SRM runs was ≤10%, demonstrating good reproducibility. Furthermore, among all the mutant SPOP peptides, FVQGKDWGVKKFIR derived from mutant SPOP^F133V^ has the best sensitivity for quantification by PRISM-SRM with the lowest LOD and LOQ values (Table 1). Interestingly, the SPOP^F133V^ mutation is the most frequently mutated residue in prostate cancer (~50% of the total SPOP mutations) [5]. Therefore, our newly developed point-mutation-specific PRISM-SRM assays could be very useful to study the most commonly observed SPOP protein mutations in prostate cancer.

**Fig. 3:**
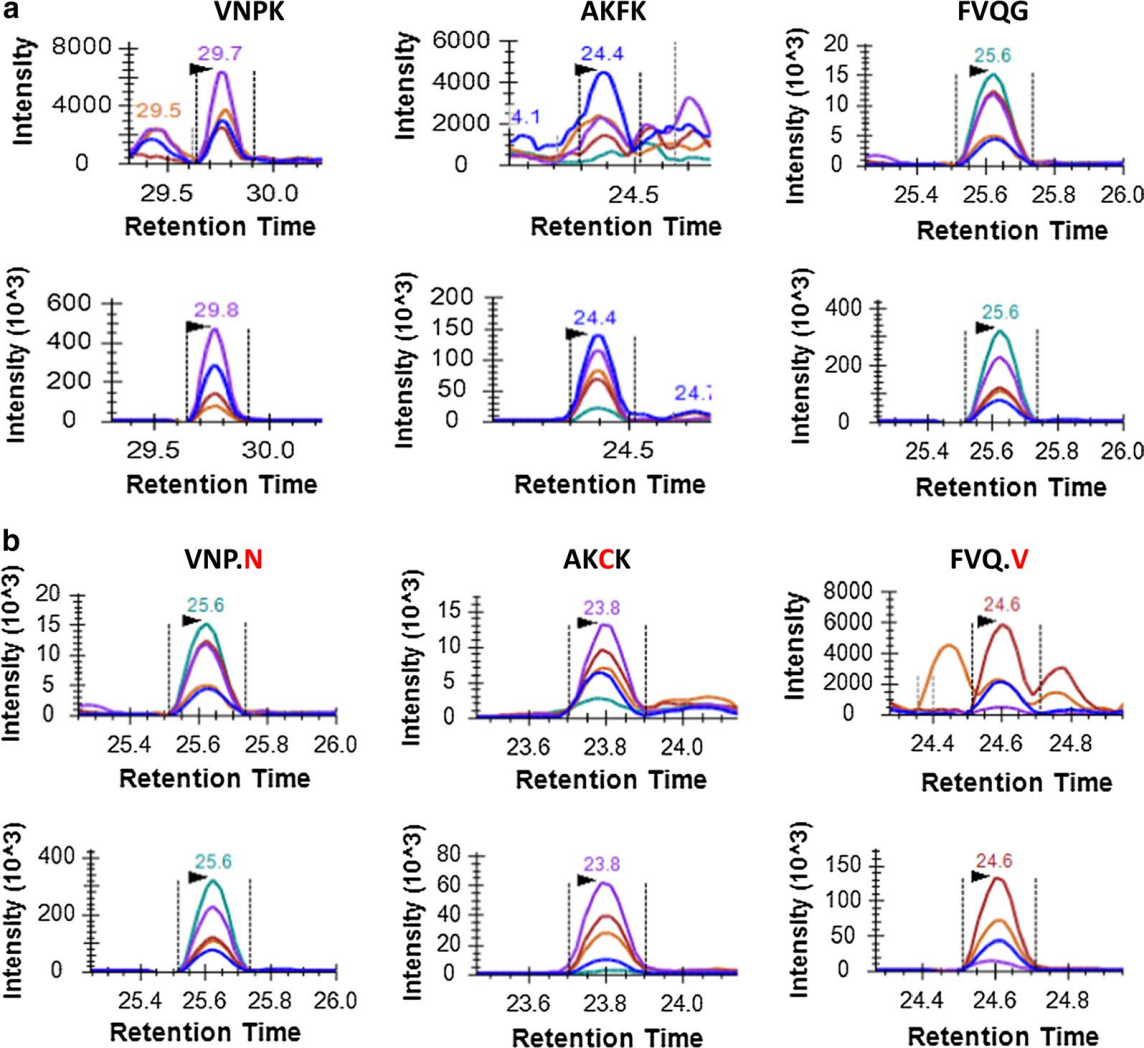
XICs of SPOP WT peptides in mutant region detected in HEK293T cell lines expressing WT SPOP cell line (**a**) and their corresponding mutant counterparts detected in SPOP Y87N, F102C and F133V mutant cell lines (**b**) using PRISM-SRM (AA shown in *red font* indicates mutation site). XICs for all the peptide candidates (details available in Additional file 4: Table S2) in four SPOP cell lines were shown in Additional file 9: Figure S7

The optimized PRISM-SRM assay was applied to quantitatively analyze protein digests from SPOP cell line samples. A total of 5 cell lines including HEK293T cells and HEK293T cells expressing either SPOP-WT or SPOP-mutant (Y87N, F102C, and F133V) were tested. The XICs of SPOP peptides in HEK293T cell lines expressing SPOP-WT and three SPOP mutations by PRISM-SRM analysis are shown in Fig. 3 and Additional file 9: Figure S7. All the endogenous peptides, both WT and mutant peptides, were detected clearly in the corresponding transfected cell line samples, but not in the non-transfected HEK293T control sample (data not shown). Compared to the LC-SRM analysis, the increased loading and reduced background interference in PRISM-SRM enabled the detection of peptides in even the cell lines with lower SPOP protein expression, such as VNPKGLDEESKDYLSLYLLLVSCPKSEVR and AKFKFSILNAKGEETKAMESQR in WT and F133V cells. The CV of peptide VNPKGLDEESKDYLSLNLLLVSCPKSEVR and AKCKFSILNAKGEETKAMESQR was calculated as 3.7 and 4.6%, respectively. The CV for the mutant peptide FVQGKDWGVKKFIR’s was 16.7% due to the lower abundance of the endogenous protein. Overall, the CV of each SPOP peptide in all samples was ≤20% showing good reproducibility of the PRISM-SRM assay.

The SPOP protein concentrations were calculated based on the peak area ratio and the response curve (Table 2). Although detected, peptide AKFKFSILNAKGEETKAMESQR in the SPOP-WT and F133V cell lines was below LOQ and could not be quantified. From the two peptides in the non-mutant region (LADELGGLWENSR and SLASAQCPFLGPPR), it can be seen that the overall expression of WT and F133V is 10–20 times lower than that of Y87N and F102C in the corresponding cell lines. The mutant peptides VNPKGLDEESKDYLSLNLLLVSCPKSEVR, AKCKFSILNAKGEETKAMESQR and FVQGKDWGVKKF in the three transfected cell lines were determined to be 61.4, 9.4 and 1.8 fmol/µg of total protein, respectively.

**Table 2:** Quantification of SPOP peptides in HEK293T cells expressing WT and mutant SPOP proteins by PRISM-SRM

Cell line	VNPKGLDEESKDYLSLYLLLVSCPKSEVR		AKFKFSILNAKGEETKAMESQR		FVQGKDWGFKKFIR		LADELGGLWENSR	SLASAQCPFLGPPR
	WT	Y87N	WT	F102C	WT	F133V	WT	WT
SPOP^WT^	1.3 ± 0.1		NQ		2.4 ± 0.3		6.2 ± 0.2	21.4 ± 0.5
SPOP^Y87N^		61.4 ± 2.3	37.6 ± 2.4		43.6 ± 2.3		180 ± 1	435 ± 9
SPOP^F102C^	98.6 ± 1.0			9.4 ± 0.6	61.1 ± 7.7		188 ± 2	468 ± 26
SPOP^F133V^	4.3 ± 0.4		NQ			1.8 ± 0.4	13.9 ± 0.03	29.1 ± 0.4

The peptide concentrations are shown in fmol/µg of total protein

*NQ* not quantified

## Discussion

Studies on protein mutations have relied heavily on isoform-specific antibodies. However, it is very challenging to develop high-quality antibodies for specific recognition of protein isoforms without cross-reactivity. Compared to antibody-based methods, MS-based targeted proteomics has higher specificity, shorter lead times and much higher success rates in developing sensitive, specific and accurate assays for protein isoform quantification. The high quality heavy isotope-labeled peptide standards needed for highly confident and precise quantification can also be obtained readily from commercial vendors. Moreover, front-end peptide enrichment methods such as SISCAPA (stable isotope standards and capture by anti-peptide antibodies) [31] and PRISM enable protein quantification at low ng/mL to 50 pg/mL levels in blood plasma/serum, rivaling the sensitivity of ELISA assays. Thus, targeted proteomics provides a valuable alternative for preclinical validation of protein isoforms (e.g., mutation, splice variant, gene fusion, and post-translational modification) as potential disease biomarkers.

The SRM quantification of mutant SPOP proteins has some unique challenges that are inherently associated with its AA sequence: there are seven potential mutation sites distributed in a 49-AA region containing eight lysine and three arginine residues. This precludes the conventional enzymatic digestion methods using trypsin or Lys-C because most of the enzymatically cleaved peptides covering the mutation site(s) are too short to be analyzed by LC–MS/MS with good specificity. Arg-C produces longer peptides that cover all the mutation sites (14–29 AA). However, these peptides are in general less responsive in electrospray ionization, compared to typical tryptic peptides that are selected for SRM analysis (e.g., 6–20 AA). The presence of multiple internal lysine residues also causes the most MS-responsive precursor ions to carry 4+ charges (note that the Asp-N peptides also have multiple internal lysine residues and in general provide even lower MS signal). This results in small differences in *m/z* of the precursor ions, hence the risk of having the mutant and WT peptides co-scanned (causing reduced specificity), as well as decreased fragmentation efficiency, because the beam-type collision in the triple quadrupole MS instrument is not as efficient as the electron transfer dissociation available on the hybrid Orbitrap instrument for fragmenting long, highly charged peptides. Moreover, these long Arg-C peptides are also highly hydrophobic, causing significant peptide loss in peptide standard handling and PRISM fractionation.

The following procedures were used to overcome these difficulties and substantially improve the assay quality: (1) high-resolution LC separation helps to fully resolve the mutant and WT peptides, eliminating potential ambiguity and interference in peptide quantification; (2) adding protein digests as a protective matrix to the pure peptide stocks effectively reduces loss of the hydrophobic Arg-C peptide internal standards; (3) use of TFA instead of the basic mobile phases in the PRISM fractionation significantly improves recovery of the SPOP peptides that have high pI values; and (4) PRISM-SRM provides higher sensitivity than regular LC-SRM for SPOP mutation analysis. As a result, all the PRISM-SRM assays showed a linear dynamic range of more than two orders of magnitude, with LOQ of 0.2–1 fmol/μg of total protein in the cell lysate for the mutation site(s)-containing peptides. In this report, the three most frequent SPOP mutations in prostate cancer, Y87N, F102C, and F133V, were unambiguously confirmed in respective transfected HEK293T cell lines using these PRISM-SRM assays. Since the SPOP mutations behave as loss of function mutants in cell line experiments [32], it will be important to determine in future experiments the relative abundance of mutant and wild type SPOP protein present in prostate tumor tissues.

## Conclusions

In summary, PRISM-SRM provides a sensitive, isoform-specific, multiplexed, and antibody-independent approach for quantification of SPOP mutations at the protein level, which holds great promise for the verification of the mutant SPOP proteins as biomarkers for prostate cancer.

## Additional files

**Additional file 1: Table S1.** Peptides resulted from in silico digestion using different proteolytic enzymes.

**Additional file 2: Figure S1.** Heat map of spectral counts of the peptides generated from recombinant SPOP protein by Arg-C and Asp-N digestion. The AAs shown in red and underlined fonts represent the reported potential mutant sites. The sequence of SPOP is obtained from UniProt (http://www.uniprot.org/).

**Additional file 3: Figure S2.** XICs of the transitions monitored for SPOP heavy peptides VNPKGLDEESKDYLSLYLLLVSCPKSEVR (A) and VNPKGLDEESKDYLSLCLLLVSCPKSEVR (B).

**Additional file 4: Table S2.** Details of the PRISM-SRM assays developed for the SPOP peptides in the mutation and non-mutation regions.

**Additional file 5: Figure S3.** XICs of SPOP peptide VNPKGLDEESKDYLSLYLLLVSCPKSEVR and its mutant variants VNPKGLDEESKDYLSLCLLLVSCPKSEVR and VNPKGLDEESKDYLSLNLLLVSCPKSEVR without (A) and with (B) protective matrix in the pure peptide stocks in LC-SRM analysis.

**Additional file 6: Figure S4.** Effect of hydrophobicity on peptide recovery (heavy/light peak area ratio of SPOP peptides without matrix versus that with matrix). SPOP peptides were ordered by their theoretical hydrophobicity values. Corresponding peptide information is listed in Additional file 4: Table S2.

**Additional file 7: Figure S5.** XICs of SPOP peptides in WT and HEK293T cell lines expressing WT and mutant SPOP (F133V) by regular LC-SRM analysis.

**Additional file 8: Figure S6.** Improvement of PRISM-SRM over LC-SRM detection of SPOP heavy peptides in the mutation region under different PRISM separation conditions: high pH (blue), 0.1% FA (red) and 0.1% TFA (green). The theoretical pIs are marked on the top of each peptide. Corresponding peptide information is listed in Additional file 4: Table S2.

**Additional file 9: Figure S7.** XICs of SPOP peptides in HEK 293T cell lines expressed by SPOP-WT (A) and mutant SPOP Y87N (B), F102C (C), and F133V (D) using PRISM-SRM analysis (marked red AA means mutant substitution). Corresponding peptide information is listed in Additional file 4: Table S2.

## Abbreviations

SPOP: speckle-type POZ protein
PRISM: high-pressure, high-resolution separations coupled with intelligent selection and multiplexing
SRM: selected reaction monitoring
MS: mass spectrometry
DTT: dithiothreitol
IAA: iodoacetamide
WT: wild type
HEK: human embryonic kidney
TFA: trifluoroacetic acid
CE: collision energy
LOD: limit of detection
LOQ: limit of quantification
XIC: extracted ion chromatogram
