# Supplementary material for: Quantification of mutant SPOP proteins in prostate cancer using mass spectrometry-based targeted proteomics
Source: J Transl Med. 2017 Aug 15;15:175. doi: 10.1186/s12967-017-1276-7 (PMC5557563; doi:10.1186/s12967-017-1276-7)
Supplement: Supplementary file 2 — Additional file 2: Figure S1. Heat map of spectral counts of the peptides generated from recombinant SPOP protein by Arg-C and Asp-N digestion. The AAs shown in red and underlined fonts represent the reported potential mutant sites. The sequence of SPOP is obtained from UniProt (http://www.uniprot.org/). [file 12967_2017_1276_MOESM2_ESM.pptx]

## Slide 1
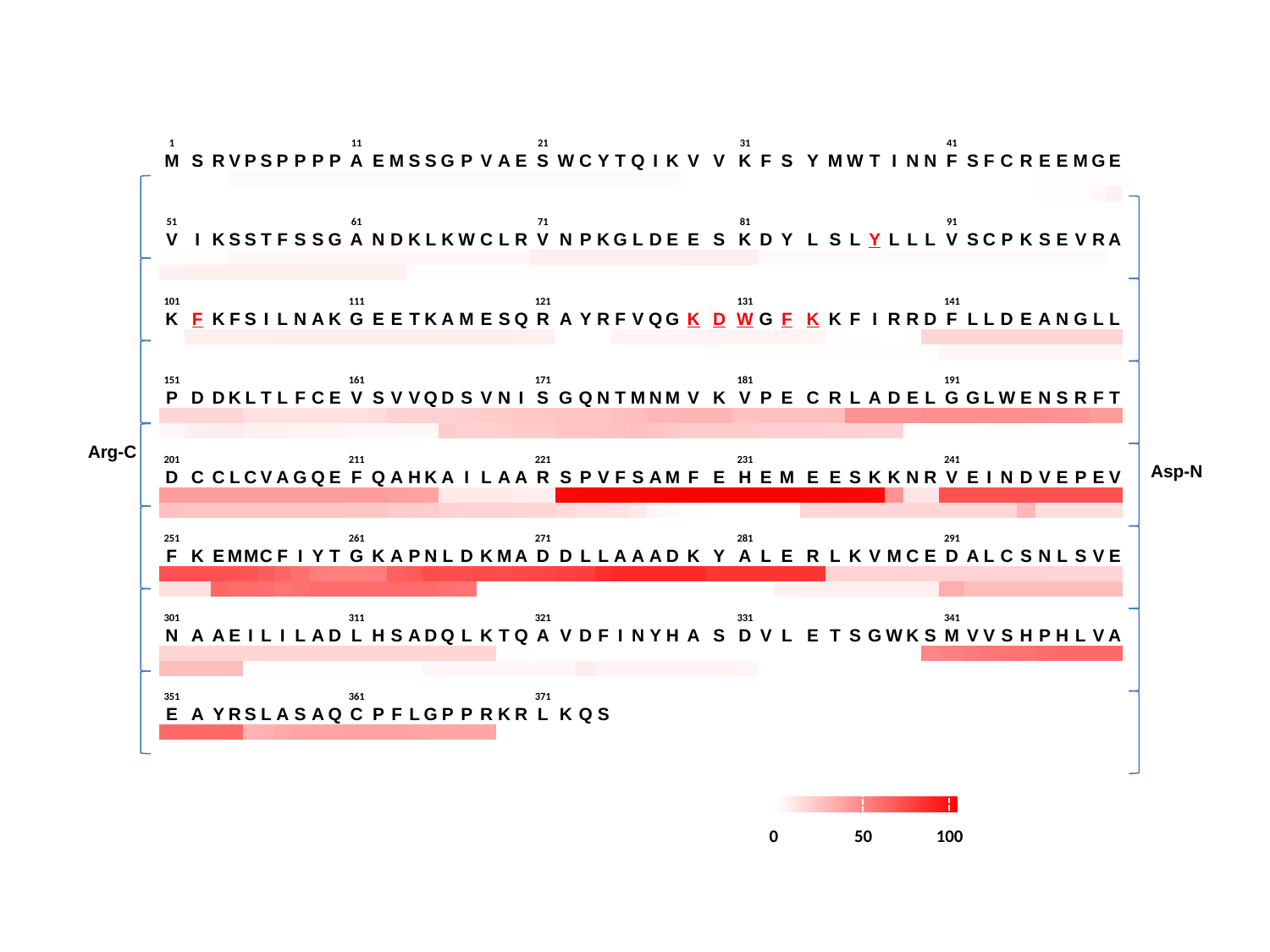

| 1 | | | | | | | | | | 11 | | | | | | | | | | 21 | | | | | | | | | | 31 | | | | | | | | | | 41 | | | | | | | | | |
| --- | --- | --- | --- | --- | --- | --- | --- | --- | --- | --- | --- | --- | --- | --- | --- | --- | --- | --- | --- | --- | --- | --- | --- | --- | --- | --- | --- | --- | --- | --- | --- | --- | --- | --- | --- | --- | --- | --- | --- | --- | --- | --- | --- | --- | --- | --- | --- | --- | --- |
| M | S | R | V | P | S | P | P | P | P | A | E | M | S | S | G | P | V | A | E | S | W | C | Y | T | Q | I | K | V | V | K | F | S | Y | M | W | T | I | N | N | F | S | F | C | R | E | E | M | G | E |
| | | | | | | | | | | | | | | | | | | | | | | | | | | | | | | | | | | | | | | | | | | | | | | | | | |
| | | | | | | | | | | | | | | | | | | | | | | | | | | | | | | | | | | | | | | | | | | | | | | | | | |
| | | | | | | | | | | | | | | | | | | | | | | | | | | | | | | | | | | | | | | | | | | | | | | | | | |
| 51 | | | | | | | | | | 61 | | | | | | | | | | 71 | | | | | | | | | | 81 | | | | | | | | | | 91 | | | | | | | | | |
| V | I | K | S | S | T | F | S | S | G | A | N | D | K | L | K | W | C | L | R | V | N | P | K | G | L | D | E | E | S | K | D | Y | L | S | L | Y | L | L | L | V | S | C | P | K | S | E | V | R | A |
| | | | | | | | | | | | | | | | | | | | | | | | | | | | | | | | | | | | | | | | | | | | | | | | | | |
| | | | | | | | | | | | | | | | | | | | | | | | | | | | | | | | | | | | | | | | | | | | | | | | | | |
| | | | | | | | | | | | | | | | | | | | | | | | | | | | | | | | | | | | | | | | | | | | | | | | | | |
| 101 | | | | | | | | | | 111 | | | | | | | | | | 121 | | | | | | | | | | 131 | | | | | | | | | | 141 | | | | | | | | | |
| K | F | K | F | S | I | L | N | A | K | G | E | E | T | K | A | M | E | S | Q | R | A | Y | R | F | V | Q | G | K | D | W | G | F | K | K | F | I | R | R | D | F | L | L | D | E | A | N | G | L | L |
| | | | | | | | | | | | | | | | | | | | | | | | | | | | | | | | | | | | | | | | | | | | | | | | | | |
| | | | | | | | | | | | | | | | | | | | | | | | | | | | | | | | | | | | | | | | | | | | | | | | | | |
| | | | | | | | | | | | | | | | | | | | | | | | | | | | | | | | | | | | | | | | | | | | | | | | | | |
| 151 | | | | | | | | | | 161 | | | | | | | | | | 171 | | | | | | | | | | 181 | | | | | | | | | | 191 | | | | | | | | | |
| P | D | D | K | L | T | L | F | C | E | V | S | V | V | Q | D | S | V | N | I | S | G | Q | N | T | M | N | M | V | K | V | P | E | C | R | L | A | D | E | L | G | G | L | W | E | N | S | R | F | T |
| | | | | | | | | | | | | | | | | | | | | | | | | | | | | | | | | | | | | | | | | | | | | | | | | | |
| | | | | | | | | | | | | | | | | | | | | | | | | | | | | | | | | | | | | | | | | | | | | | | | | | |
| | | | | | | | | | | | | | | | | | | | | | | | | | | | | | | | | | | | | | | | | | | | | | | | | | |
| 201 | | | | | | | | | | 211 | | | | | | | | | | 221 | | | | | | | | | | 231 | | | | | | | | | | 241 | | | | | | | | | |
| D | C | C | L | C | V | A | G | Q | E | F | Q | A | H | K | A | I | L | A | A | R | S | P | V | F | S | A | M | F | E | H | E | M | E | E | S | K | K | N | R | V | E | I | N | D | V | E | P | E | V |
| | | | | | | | | | | | | | | | | | | | | | | | | | | | | | | | | | | | | | | | | | | | | | | | | | |
| | | | | | | | | | | | | | | | | | | | | | | | | | | | | | | | | | | | | | | | | | | | | | | | | | |
| | | | | | | | | | | | | | | | | | | | | | | | | | | | | | | | | | | | | | | | | | | | | | | | | | |
| 251 | | | | | | | | | | 261 | | | | | | | | | | 271 | | | | | | | | | | 281 | | | | | | | | | | 291 | | | | | | | | | |
| F | K | E | M | M | C | F | I | Y | T | G | K | A | P | N | L | D | K | M | A | D | D | L | L | A | A | A | D | K | Y | A | L | E | R | L | K | V | M | C | E | D | A | L | C | S | N | L | S | V | E |
| | | | | | | | | | | | | | | | | | | | | | | | | | | | | | | | | | | | | | | | | | | | | | | | | | |
| | | | | | | | | | | | | | | | | | | | | | | | | | | | | | | | | | | | | | | | | | | | | | | | | | |
| | | | | | | | | | | | | | | | | | | | | | | | | | | | | | | | | | | | | | | | | | | | | | | | | | |
| 301 | | | | | | | | | | 311 | | | | | | | | | | 321 | | | | | | | | | | 331 | | | | | | | | | | 341 | | | | | | | | | |
| N | A | A | E | I | L | I | L | A | D | L | H | S | A | D | Q | L | K | T | Q | A | V | D | F | I | N | Y | H | A | S | D | V | L | E | T | S | G | W | K | S | M | V | V | S | H | P | H | L | V | A |
| | | | | | | | | | | | | | | | | | | | | | | | | | | | | | | | | | | | | | | | | | | | | | | | | | |
| | | | | | | | | | | | | | | | | | | | | | | | | | | | | | | | | | | | | | | | | | | | | | | | | | |
| | | | | | | | | | | | | | | | | | | | | | | | | | | | | | | | | | | | | | | | | | | | | | | | | | |
| 351 | | | | | | | | | | 361 | | | | | | | | | | 371 | | | | | | | | | | | | | | | | | | | | | | | | | | | | | |
| E | A | Y | R | S | L | A | S | A | Q | C | P | F | L | G | P | P | R | K | R | L | K | Q | S | | | | | | | | | | | | | | | | | | | | | | | | | | |
| | | | | | | | | | | | | | | | | | | | | | | | | | | | | | | | | | | | | | | | | | | | | | | | | | |
| | | | | | | | | | | | | | | | | | | | | | | | | | | | | | | | | | | | | | | | | | | | | | | | | | |
| | | | | | | | | | | | | | | | | | | | | | | | | | | | | | | | | | | | | | | | | | | | | | | | | | |
Arg-C
Asp-N
0 50 100
