# Supplementary material for: Quantification of mutant SPOP proteins in prostate cancer using mass spectrometry-based targeted proteomics
Source: J Transl Med. 2017 Aug 15;15:175. doi: 10.1186/s12967-017-1276-7 (PMC5557563; doi:10.1186/s12967-017-1276-7)
Supplement: Supplementary file 3 — Additional file 3: Figure S2. XICs of the transitions monitored for SPOP heavy peptides VNPKGLDEESKDYLSLYLLLVSCPKSEVR (A) and VNPKGLDEESKDYLSLCLLLVSCPKSEVR (B). [file 12967_2017_1276_MOESM3_ESM.pptx]

## Slide 1
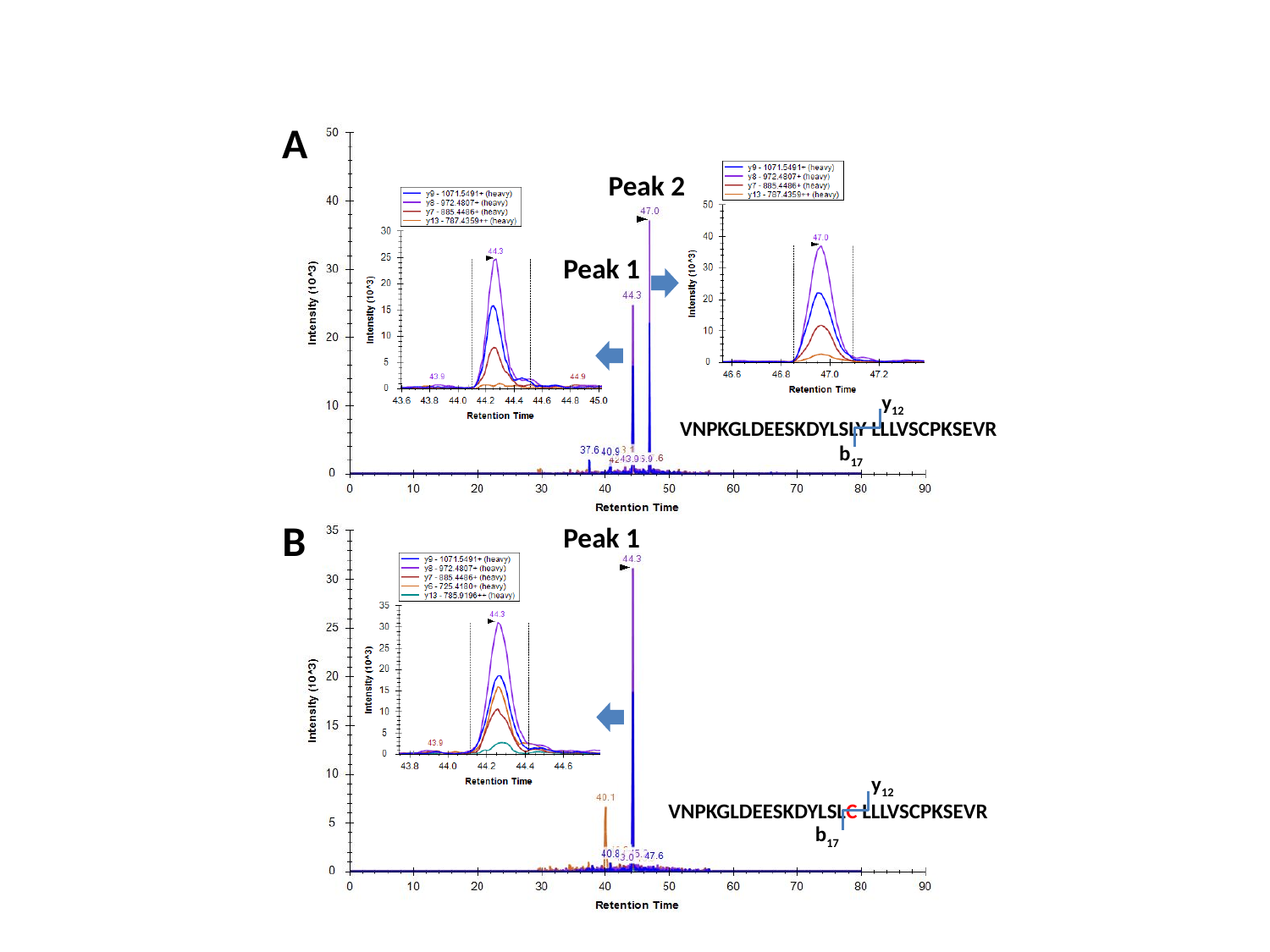

A
Peak 2
Peak 1
y12
VNPKGLDEESKDYLSLY LLLVSCPKSEVR
b17
B
Peak 1
y12
VNPKGLDEESKDYLSLC LLLVSCPKSEVR
b17
