# Supplementary material for: Quantification of mutant SPOP proteins in prostate cancer using mass spectrometry-based targeted proteomics
Source: J Transl Med. 2017 Aug 15;15:175. doi: 10.1186/s12967-017-1276-7 (PMC5557563; doi:10.1186/s12967-017-1276-7)
Supplement: Supplementary file 5 — Additional file 5: Figure S3. XICs of SPOP peptide VNPKGLDEESKDYLSLYLLLVSCPKSEVR and its mutant variants VNPKGLDEESKDYLSLCLLLVSCPKSEVR and VNPKGLDEESKDYLSLNLLLVSCPKSEVR without (A) and with (B) protective matrix in the pure peptide stocks in LC-SRM analysis. [file 12967_2017_1276_MOESM5_ESM.pptx]

## Slide 1
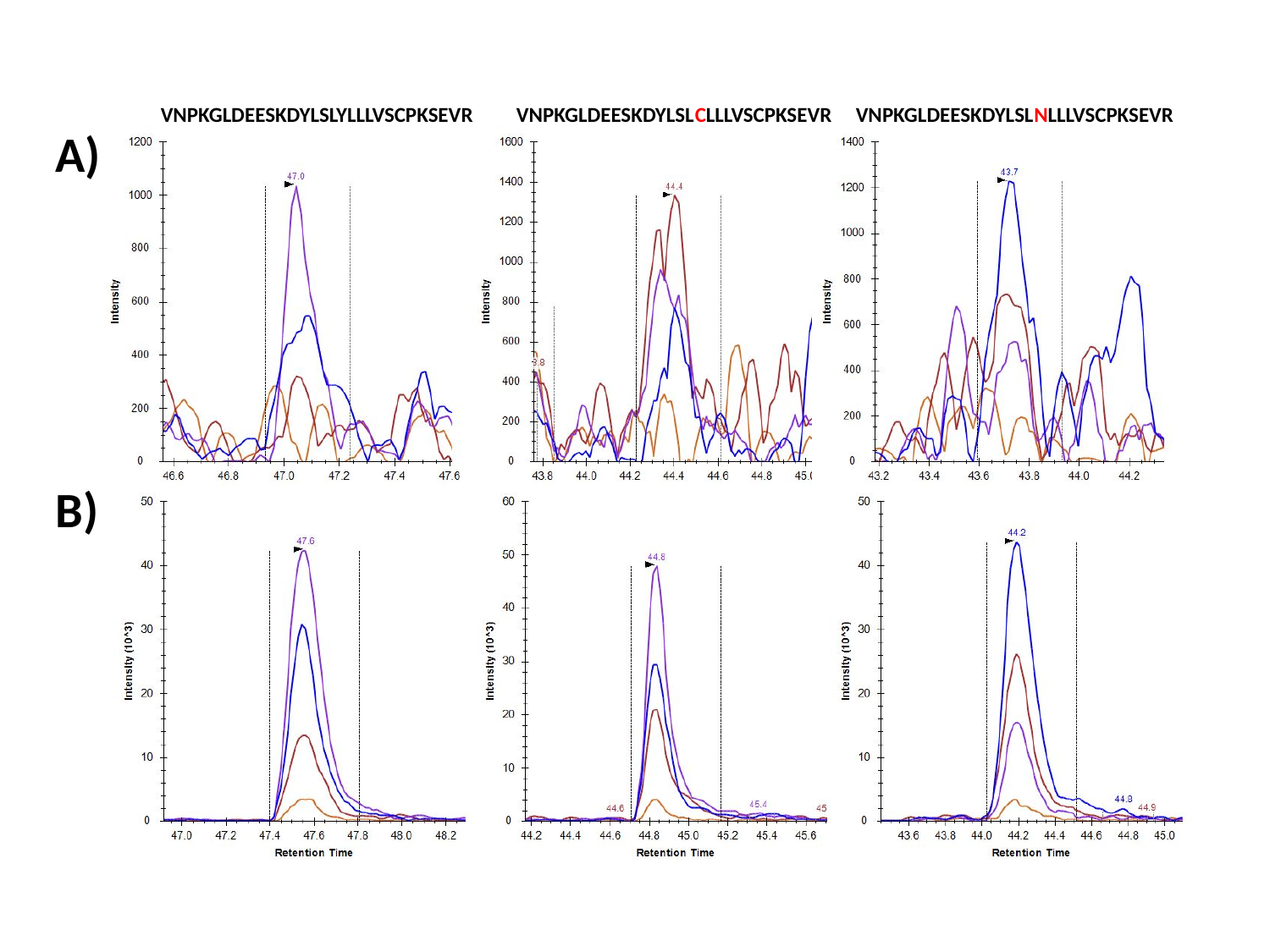

VNPKGLDEESKDYLSLYLLLVSCPKSEVR VNPKGLDEESKDYLSLCLLLVSCPKSEVR VNPKGLDEESKDYLSLNLLLVSCPKSEVR
A)
B)
