# Supplementary figures and images for: Quantification of mutant SPOP proteins in prostate cancer using mass spectrometry-based targeted proteomics
Source: J Transl Med. 2017 Aug 15;15:175. doi: 10.1186/s12967-017-1276-7 (PMC5557563; doi:10.1186/s12967-017-1276-7)

## Slide 1
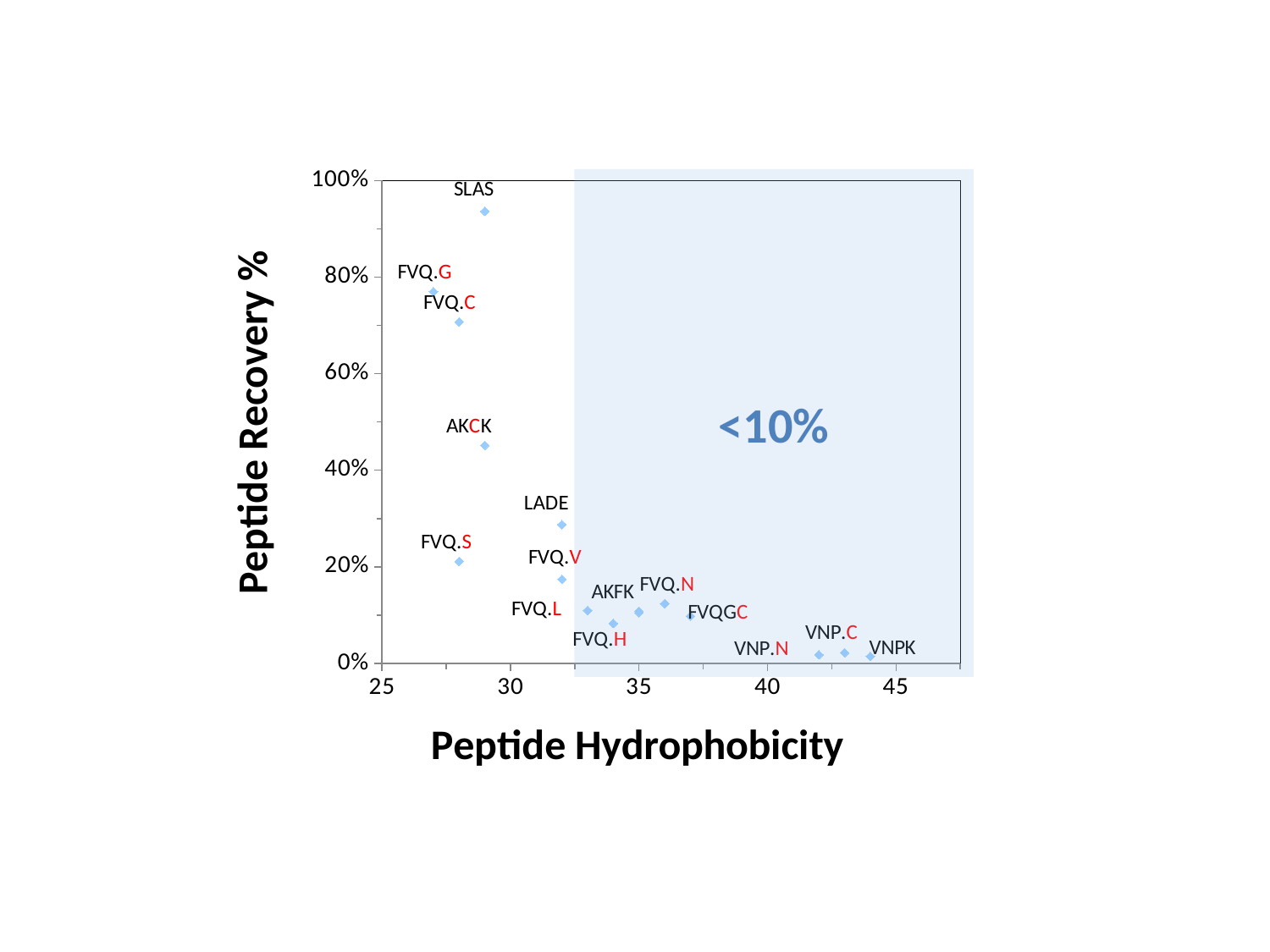

### Chart
| Category | |
|---|---|<10%

Supplement: Supplementary file 6 — Additional file 6: Figure S4. Effect of hydrophobicity on peptide recovery (heavy/light peak area ratio of SPOP peptides without matrix versus that with matrix). SPOP peptides were ordered by their theoretical hydrophobicity values. Corresponding peptide information is listed in Additional file 4: Table S2. [file 12967_2017_1276_MOESM6_ESM.pptx]

## Slide 1
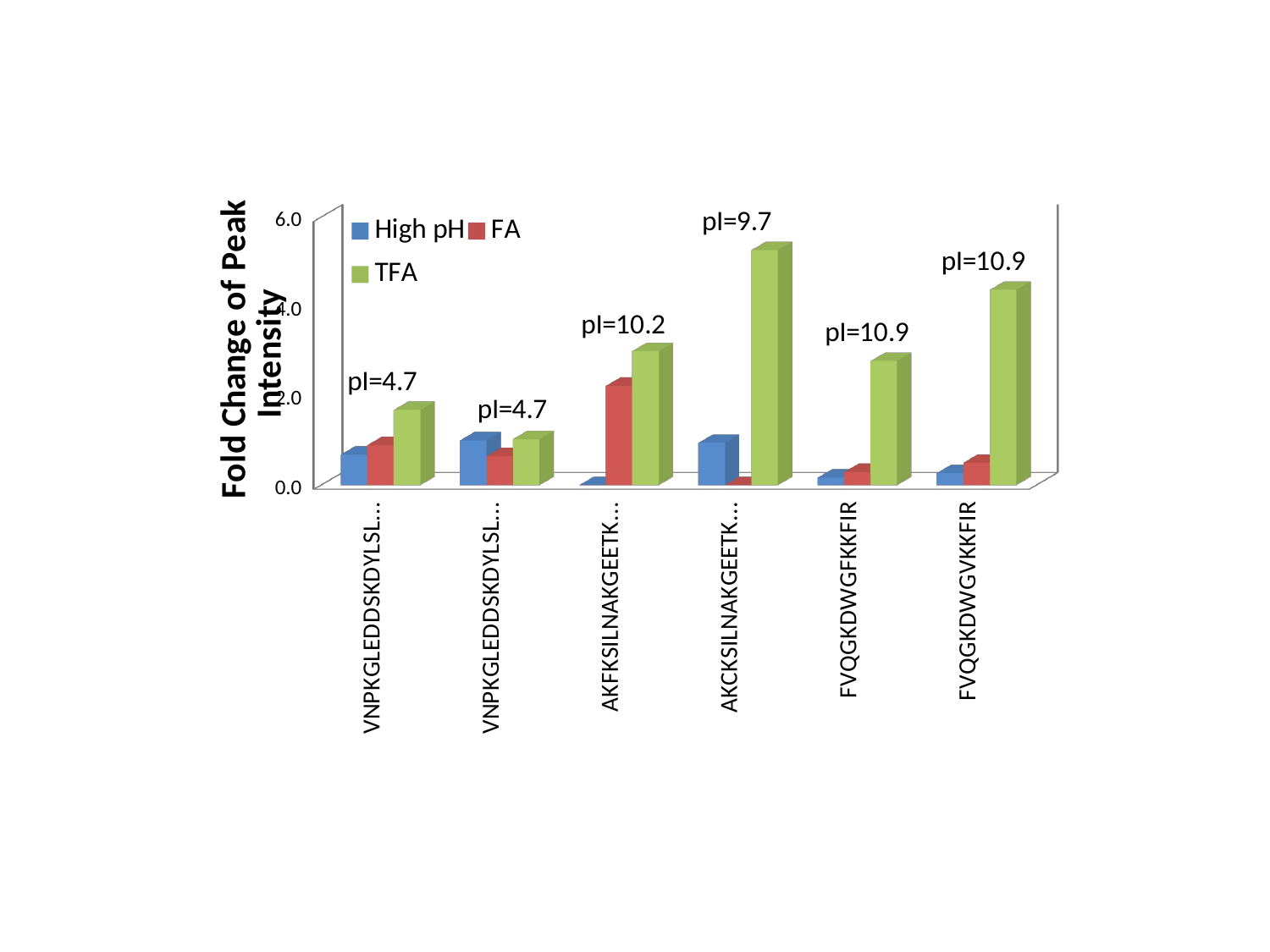

[unsupported chart]

Supplement: Supplementary file 8 — Additional file 8: Figure S6. Improvement of PRISM-SRM over LC-SRM detection of SPOP heavy peptides in the mutation region under different PRISM separation conditions: high pH (blue), 0.1% FA (red) and 0.1% TFA (green). The theoretical pIs are marked on the top of each peptide. Corresponding peptide information is listed in Additional file 4: Table S2. [file 12967_2017_1276_MOESM8_ESM.pptx]
