# Supplementary material for: Quantification of mutant SPOP proteins in prostate cancer using mass spectrometry-based targeted proteomics
Source: J Transl Med. 2017 Aug 15;15:175. doi: 10.1186/s12967-017-1276-7 (PMC5557563; doi:10.1186/s12967-017-1276-7)
Supplement: Supplementary file 7 — Additional file 7: Figure S5. XICs of SPOP peptides in WT and HEK293T cell lines expressing WT and mutant SPOP (F133V) by regular LC-SRM analysis. [file 12967_2017_1276_MOESM7_ESM.pptx]

## Slide 1
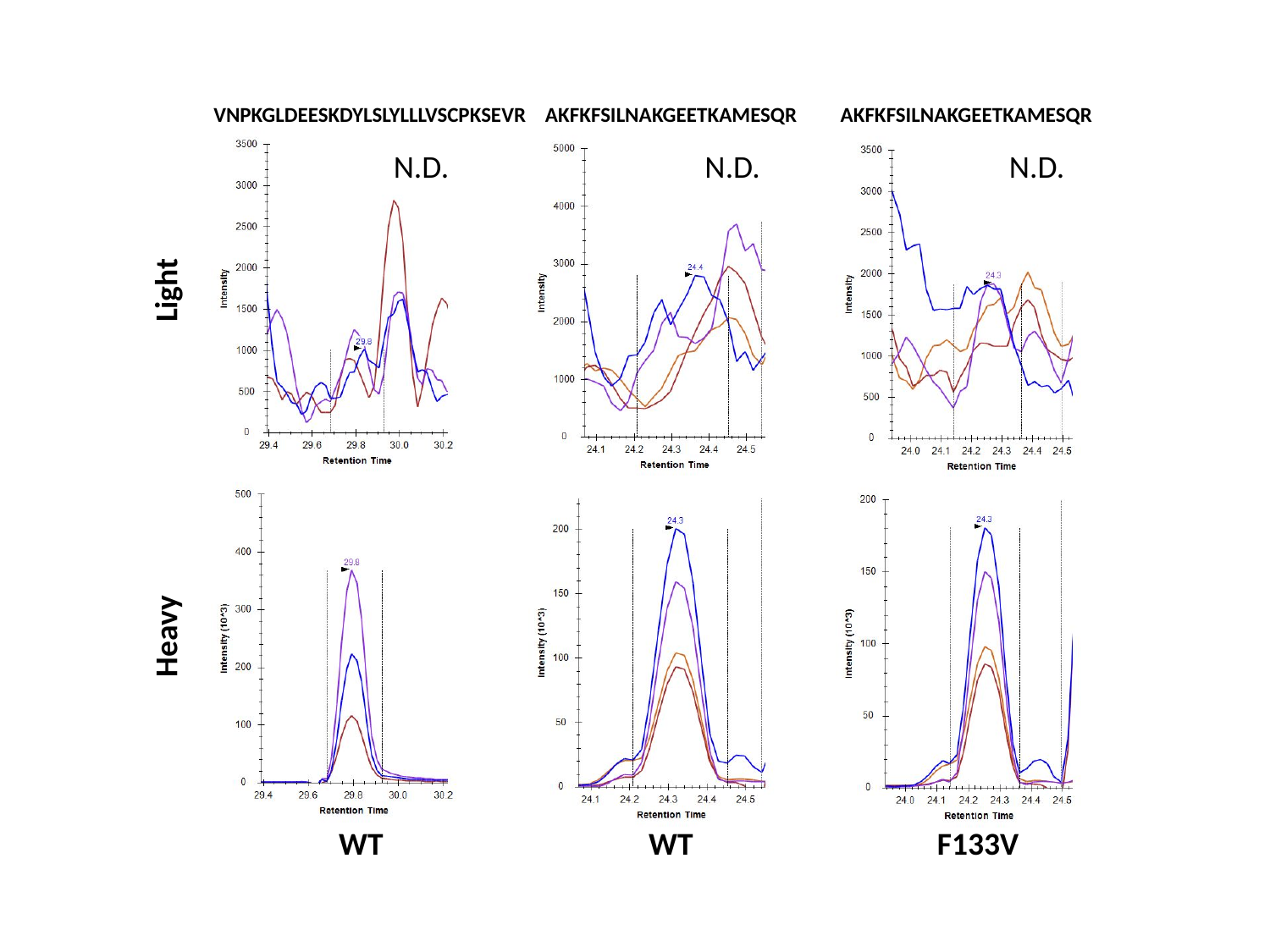

VNPKGLDEESKDYLSLYLLLVSCPKSEVR AKFKFSILNAKGEETKAMESQR AKFKFSILNAKGEETKAMESQR
N.D.		 N.D.		 N.D.
Heavy Light
WT WT F133V
