# Supplementary material for: Quantification of mutant SPOP proteins in prostate cancer using mass spectrometry-based targeted proteomics
Source: J Transl Med. 2017 Aug 15;15:175. doi: 10.1186/s12967-017-1276-7 (PMC5557563; doi:10.1186/s12967-017-1276-7)
Supplement: Supplementary file 9 — Additional file 9: Figure S7. XICs of SPOP peptides in HEK 293T cell lines expressed by SPOP-WT (A) and mutant SPOP Y87N (B), F102C (C), and F133V (D) using PRISM-SRM analysis (marked red AA means mutant substitution). Corresponding peptide information is listed in Additional file 4: Table S2. [file 12967_2017_1276_MOESM9_ESM.pptx]

## Slide 1
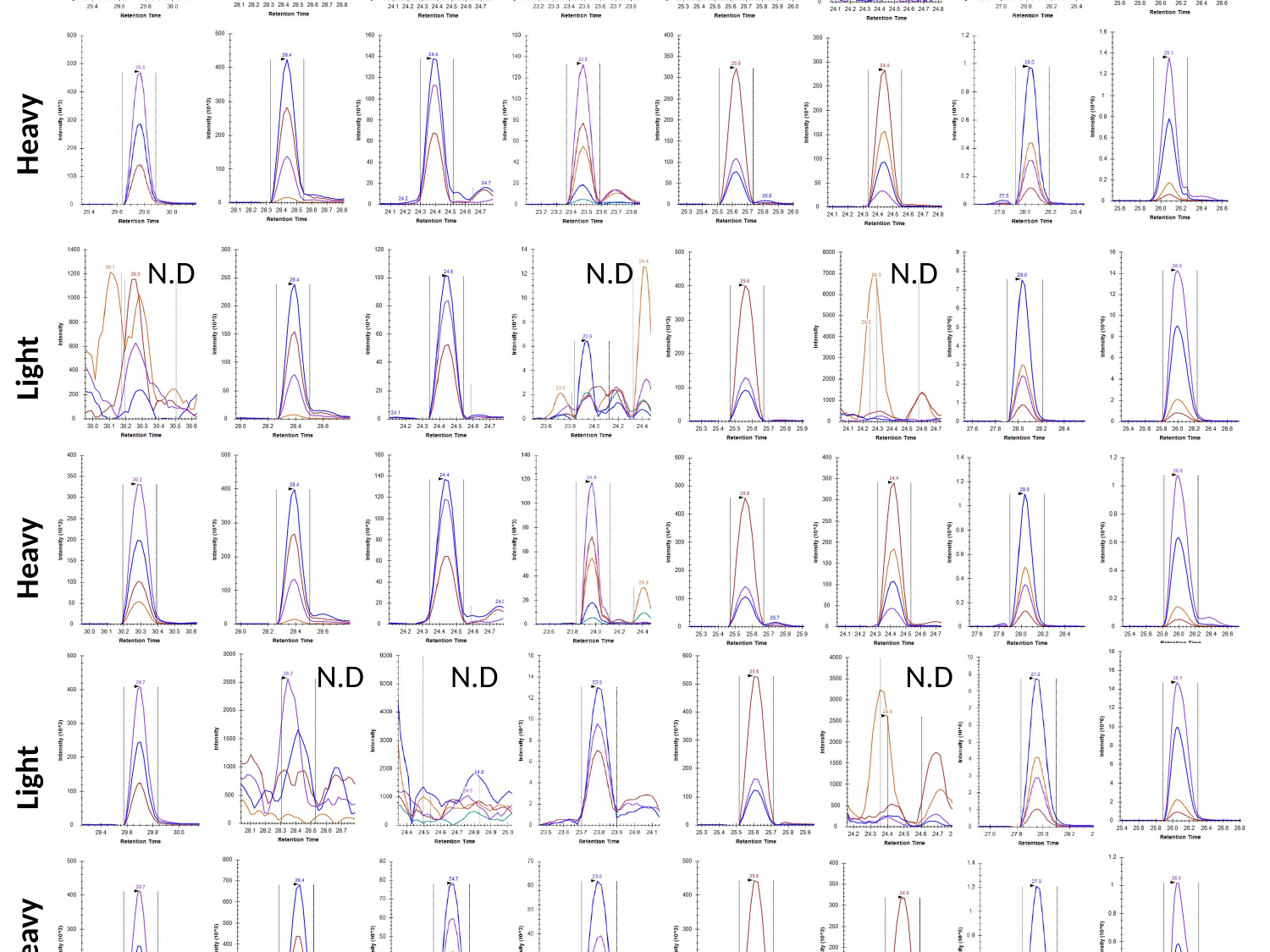

VNPK VNP.N AKFK AKCK FVQG FVQ.V LADE SLAS
N.D		 N.D		 N.D
 WT
 Y87
 F102
 F133
N.D		 	 N.D		 N.D
Heavy 	 Light		Heavy 	 Light		Heavy 	 Light	 Heavy 	Light
N.D	 N.D		 N.D
N.D	 N.D	 N.D
